# Searching for New Model of Digital Informatics for Human–Computer Interaction: Testing the Institution-Based Technology Acceptance Model (ITAM)

**DOI:** 10.3390/ijerph18115593

**Published:** 2021-05-24

**Authors:** Youngcheoul Kang, Nakbum Choi, Seoyong Kim

**Affiliations:** 1 Graduate School of Public Administration, Korea University, Sejong 30019, Korea; paschal814@gmail.com; 2 Department of Police Administration, Seowon University, Cheongju 28674, Korea; 3 Department of Public Administration, Ajou University, Suwon 16499, Korea; seoyongkim@ajou.ac.kr

**Keywords:** new information technology, Technology Acceptance Model (TAM), institution, institutionalism, digital informatics, human–computer interaction

## Abstract

The fourth industrial revolution has produced new information technology (IT) that is widely used in the healthcare industry. Although the nature of the institution affects IT adoption, the Technology Acceptance Model (TAM), a dominant theory, has dismissed its role and influence. Our research investigates how institutions influence the adoption of new IT by using the Institution-based Technology Acceptance Model (ITAM). We conducted an empirical test by using survey data collected from 300 employees in the public sector. Structural equation modeling was applied to test the proposed hypotheses. The results showed the total effect of institutions on the intention to use new IT is positive and significant. Second, IT adoption is not only affected by external institutions but also by type of institution; the external institution takes a greater role in inducing perceived usefulness, perceived ease of use, and intention to use than does the internal. Third, perceived ease of use and perceived usefulness play mediating roles between institution and IT adoption. Fourth, an alternative expanded model to which more individual and organizational factors were added confirmed the results of the base model. We concluded that institutions have a strong impact on the level of intention for IT use through perceived ease of use and perceived usefulness.

## 1. Introduction

The rapid development of digital technologies has led to many ideas and attempts to change developmental paths in society [1]. These recent developments, along with the advancement of social media tools and IT, provide innovative and intense motivations to strengthen interactions between organizational boundaries and individuals [2,3]. Some argue that new technological developments could pave the way for long-term growth [4]. The development of digital technologies transforms conventional industry manufacturing, service delivery, policy decision-making, public participation and also influences the digital health management related to public health. The force of technologies leads to innovative approaches in many spheres of society, which motivates many companies and governments to follow these technological advancements. Because recent IT growth has been based on digital data availability and interconnections among individuals, every person has their own preference for using IT platforms, which require extensive resources to set up [2,5,6,7]. Thus, it is necessary to use new technology that guarantees organizational process optimization through interactive communications with various stakeholders. It is believed that these technological progresses could facilitate sustainable development since they afford many stakeholders real-time opportunities to voice their grievances on day-to-day policy issues in their community; moreover, these developments advance digital data integration [3]. As its development could enhance technological usability, economic wealth, and political participation, new technology has become a major social and economic vehicle to change the ways people work, learn, and socialize [8]. With advancing developments of digital data, IT could lead to a break with precedent and prompt remarkable productivity and economic growth.

Applications of the fourth industrial revolution are considered to be revolutionizing structural elements in current society and have garnered attention in many countries [9]. As the scale and scope of change are vast and diverse, the impact of these new interactive technological developments is immense. The innovative development and diffusion of the fourth industrial revolution in the business sector is occurring faster than expected and many countries are trying to keep up with the pace of change. These changes are based on the idea that digital data sharing and interconnectedness among manufacturing stakeholders open new aspects of availability of technology, and many of these digitized devices will provide new approaches to acquiring individual demands and connecting to suppliers more conveniently [1]. Digitalization is becoming a major theme of development in every aspect as it provides a fundamental basis for recent technological innovations [9,10,11,12]. The fourth industrial revolution comprises new and transformative technologies, such as the internet of things, cloud computing, big data analytics, and automotive robotics, which can accelerate the pace of change in society.

Digital innovation is changing the fundamental structure of the public sector. Therefore, innovation strategies that consider the specific contexts and characteristics that exist in the public sector are necessary. Kromidha and Córdoba-Pachón ([13], p. 16) effectively explained the meaning of digital technologies in the public sector through adopting discursive institutionalism; digital innovation projects in the public sector are important strategic points of interaction for multifaceted stakeholders whose ideas and discourses could converge. In a transition context, coordinate discourse dominates thorough transformation projects, communicative discourse is valuable for change, participation in discourse is influenced by the power position, and the leadership philosophies’ value-led discourses are tools to justify desirable change. Moreover, Ashok et al. [14] empirically showed that when adopting innovative technology, organizational culture and transformational leadership traits positively affect the adoption of knowledge management, which has a positive effect on organizational culture to counter organizational inertia. 

In order to succeed in the innovation of information and communication technology in the public domain, it is important to overcome the inertia and failure factors that exist inside. Hannan and Freeman [15] define inertia from a population ecology perspective as a persistent organizational resistance to changing architectural features. If there is inertia, management requires only minimal discretion to adapt to environmental changes. To overcome this inertia, an organization must have the ability to quickly respond to new opportunities, which presumably competes with the ability to execute consistently and accurately. Moreover, Leonard-Barton [16] found that four core capabilities—employee knowledge and skills, technical systems, managerial systems, and the values and norms—have paradoxical attributions that facilitate and block innovation. Frost [17] classified the failure factors in knowledge management implementation into causal factors (lack of performance indicator, inadequate skill of knowledge managers and workers, problems with organization culture) and resultant failure culture (lack of widespread contribution, overemphasis on formation learning, and improper implementation of technology). How does one overcome those inertia and failure factors in public sectors? Hannan and Freeman [15] stressed continued learning to adapt to the new environment. Moreover, Leonard-Barton [16] argued that to succeed in new technology innovation, one must manage the paradox of conflicting capabilities. To overcome inertia in public sectors, Ashok et al. [14] stressed organizational culture and transformational leadership in knowledge management adoption. All of these are closely related to the institutional context.

Therefore, it is necessary to consider the impact of institutions and the role of governments as key factors to accelerate the usefulness of new technology and regulate basic principles. Governments are positioned to increase data availability and usage and public employees play a critical role for innovation in government [18,19,20]. Consequently, understanding the attitudes and behavioral intentions of public employees is vital for the successful adoption and implementation of new technology. The fourth industrial revolution has diverse focus areas [21] because it includes various fields related to advanced IT developments and includes digitalization in the workplace and utilization of digital interactions in community activities [21]. As a result, the fourth industrial revolution is characterized as a collection of technologies that incorporate numerous digital transformative approaches in both the physical and biological spheres, particularly in fields such as public health [21,22]. This study is especially concerned with the examination of the relationship between institutions and the adoption of the fourth industrial revolution in the public sector.

In the new information age, new theoretical concepts and models related to informatics are required. In the literature regarding new technology adoption, the Technology Acceptance Model (TAM) has been widely used as a theoretical framework for predicting individuals’ acceptance of technology. Since the TAM was introduced by Davis [23], great theoretical progress has been made by adding more contextual variables regarding individuals’ behavioral intention to use technology [23,24]. TAM has been applied in many IT studies and has received substantial theoretical and empirical support from many researchers. Thus, TAM has the practical advantage of being extended to diverse contexts and settings. Therefore, we will base our argument on the idea of TAM that posits individual adoption of technology is mostly determined by two perceptions: usefulness and ease of use. In addition, we examine how these two core variables predict individuals’ decision-making with regard to adopting new technology in institutional settings. However, the TAM model overlooks the macro-level structure and system in that it mainly focuses on the individuals’ utility from the information system at the micro-level. Human–computer interactions differ depending on not only the direct relationship between the two but also the intervening variables. Therefore, it is necessary to consider structural variables at the macro level in TAM. In this study, we examine how not only micro-level individuals’ utility but also macro-level institutional factors affect the acceptance of new technology and extend knowledge on technology adoption behaviors. We believe our findings can contribute to the extant literature of this domain and to understand critical factors when adopting new technology.

Because IT has been extensively used in the health field, it is very important to understand the fundamental structure of technology acceptance. Technology acceptance will be critical for providing better healthcare services through online IT, especially during pandemics like COVID-19. Although new technologies help healthcare services through telemedicine and online databases, some barriers to broader adoption of new technology persist. In particular, institutional readiness and the adoption of new technology by employees are necessary. Since healthcare service in nature is a system of combining micro-level individual actors and macro-level intuitional structures, the acceptance of new digital technology for health care service should consider not only humane side factors but also institutional settings which constrain people’s attitude and perception. Digital technologies, the core of new technologies, cannot operate in isolation and require integrated institutional systems. In particular, digital data sources need to be integrated and interoperable into broader institutional settings. On the other hand, the government is one of the main actors in the institutional setting for healthcare services. The use and analysis of this data heavily depends on digital infrastructure and strong support from the public health system by governments. Governments’ responsibility and strategic foresight are especially important in high-risk situations such as a pandemic. In this regard, it is necessary to understand the adoptive mechanism of new technology in the public sector, especially focusing on the impact of institutions. Therefore, our study is expect to contribute to understanding IT services for the healthcare field.

The remainder of this study is divided into four sections. First, based on previous research, the authors propose theoretical reviews and hypotheses on technology adoption behaviors of employees and focus on the relationships with institutions and TAM, in particular. The next section discusses the methodology of this study, the sample and data used, and the variables and measurement. Next, the results are presented in the following section to show the relationships among factors affecting technology adoptions. The final section discusses the implications and limitations of the study with a conclusion.

## 2. Theoretical Background and Hypotheses

### 2.1. New Information Technology in the Fourth Industrial Revolution and Its Adoption

The increased and intensive use of IT requires governments to develop IT-based tools to directly deliver public service. The recent technological developments of the fourth industrial revolution emphasize the interconnectivity and reciprocity of the digital revolution and can lead to transformational changes at a societal level [25].

While recent IT developments bring many benefits, it is necessary to recognize the challenges of these new technologies in the public sector. The advantages of implementing new technologies are immeasurable, especially in terms of assisting public organizations in improving their tasks. The new technologies have high usability and flexibility and provide high-performance service delivery at low cost [26]. However, there are challenges regarding security, privacy, trust, and lack of understanding [26]. New technology adoption can create structural inertial pressures within organizations [14]. As March [27] suggested, internal organizational change was difficult to manipulate. Since the administrative system regulates resources, any external or internal institutional forces can cause resistance on adopting the new technical systems [15]. Also, when technological innovation enters an organization, the administration should consider how to recognize and manage the acceptable behavior of employees [16]. Therefore, considering the impact of institutions at the initial stage of technology diffusion, it is crucial to examine the employees’ perception and contextual understanding of technology adoption for the success of new technology [17]. IT development provides innovative and intense contingencies that allow societies to strengthen their positions using IT platforms [2,5,6,7]. Among these trends, there have been intense and innovative attempts to reshape structures and improve performance in the public sector using interactive technology. For example, confronted with the current COVID-19 pandemic in many countries, we recognize the potential role of new technology in the fourth industrial revolution in alleviating and resolving issues [28]. However, the usage of this technology is still a long way off and each country has different institutional arrangements and resources to utilize the benefits of new technology.

With the unprecedented efforts to utilize digital technologies in the field of government and work processes, the advent of the fourth industrial revolution ushers people into digital transformation in all areas of their personal lives. Due to advanced internet technologies, this movement mainly includes ubiquitous data communication, managerial flexibility, disruptive innovations, and autonomous systems [29]. The current fourth industrial revolution movement involves the distribution and implementation of new technologies in accordance with digital interconnectivity. Many countries also plan to launch industry programs for the new technologies and have created intelligent technical stimuli to associate key actors with their implementation, including business, university, and government counterparts. For example, as initiated in 2006 by the German federal government, the fourth industrial revolution mainly presents a strategic vision and goals of economic and social transformation through digital innovation. Represented as high-tech strategies and innovation, the key distinction of the fourth industrial revolution is its participatory decision-making processes involving multiple stakeholders [30]. Along with these movements, the German government also launched an IT-related government framework that emphasizes the role of government as a facilitator to construct sustainable digital infrastructure for the IT industry.

Many other countries also made strategic preparations to support the implementation of a wide range of innovations of the fourth industrial revolution. For example, Canada launched “Digital Canada”, which includes five critical categories in new technology: connection, protection, cybersecurity, economic opportunities, and digital government [31,32,33]. Likewise, the Korean central government has tried to establish broad strategies to establish a universal digital platform [34,35,36,37]. To consider establishing a platform that can develop a network among related stakeholders involved in the fourth industrial revolution, the Presidential Committee for the fourth industrial revolution was launched in 2017 to bolster government support for related businesses [11,36]. In particular, South Korea effectively halted COVID-19 transmission during the pandemic’s early period. It was about twice as effective as the U.S. The key to South Korea’s success mainly came from blending technology and physical testing, institutional adjustment for centralized control, and coordination between actors. As a result, we concluded that South Korea is an appropriate location for studying the fourth industrial revolution’s new technology adoption.

### 2.2. Technology Acceptance Model (TAM) and Its Limitation

Any innovative or transformative movement in large organizations, especially governments, should consider many relevant factors in their implementation. Even though the significant factors of IT implementation in government are diverse and complicated, they could be categorized as technological, structural, and behavioral factors [38,39,40].

Among the causal factors for successful application, institutions play a significant role when policymakers try to frame and introduce an IT policy [41,42,43]. The diffusion of technological innovation is up to actual users such as employees, stakeholders, and citizens, who are directly influenced by institutional arrangements. Institutional structures usually include legislative frameworks, managerial guidance, administrative rules, procedural standards, and conventional decision-making procedures. With this background, we seek to understand the behavioral factors of employees on new technology adoption and development in the context of the application of the fourth industrial revolution in the South Korean government. Because of its integrative and systemic approach toward a digitalized society, the government’s support and drives are critical for successful implementation [10].

TAM has been continuously studied to estimate users’ acceptance and behaviors; its application has expanded [24]. As a theoretical extension of the theory of reasoned action (TRA) [44], TAM provides a unified theory of technology acceptance and usage and persuasive explanations for individual motives for utilizing IT and its adoption [45,46]. TAM states that two fundamental perceptions determine technology acceptance behavior: perceived ease of use and usefulness. Perceived usefulness is defined as “the extent to which a person believes that using IT will enhance their performance” ([24], p. 187). Perceived ease of use is defined as “the degree to which a person believes that using an IT will be free of effort” ([24], p. 187). Therefore, TAM is recognized as a stable and manageable framework to understand user acceptance of new technologies in various organizations [47].

TAM has been continuously revised and new variables have been added to increase its theoretical explanatory power. Davis [23]’s original research showed that TAM fully explained the impact of system characteristics on users’ behavior. However, this model only accounted for 36% of the variance and thus needed additional variables to increase the explanatory power. Dishaw and Strong [48] suggest an integration of TAM and task-technology fit structures in this direction. They argue that a more integrated model will contribute to explaining more choices regarding IT use. Venkatesh and Davis [24,49] showed that the extended model accounted for 40–60% of the variance in usefulness perceptions and 34–52% of the variance in use intentions. In particular, they found a significant role of both social influence processes, e.g., subjective norm and cognitive instrumental processes, e.g., job relevance. Lucas and Spitler [50] demonstrated that organizational variables such as social norms and the characteristics of job are more important in influencing use of the technology than users’ perceptions about the technology.

However, TAM has several limitations. First, TAM overlooks various preferences because it emphasizes individual utility. According to Chtourou and Souiden [51], while the utility aspect of TAM is important, the hedonic aspect should be considered. The results suggest that product designers should develop interfaces and products that not only satisfy utilitarian needs but also hedonic and enjoyment motivations. This requires considering not only utility at the individual level but also the variables of the individual ideal. Moreover, Beglaryan et al. [52] argued that TAM research ignores factors beyond the individual, including group and social processes related to IT implementation and technology’s organizational and social consequences.

Second, there are objective variables, not just subjective, among the causal factors affecting technology acceptance. On the other hand, TAM disregards these objective variables at the initial stage of development. Since previous research had focused on the variables existing inside the model, it overlooked the external variables. For example, Hu et al. [53] and Davis [23,54] did not consider external variables in TAM. However, recently, as interest in objective variables has increased, research on external variables has increased. Venkatesh et al. [55] focused on facilitating conditions as objective factors in the environment that an individual agrees to make an act easy to accomplish. Moreover, Agarwal and Prasad [56] tested their significant role with regard to technology, tenure in the workforce, level of education, and prior similar experiences. Igbaria et al. [57] investigated internal computing support, internal computing training, management support, external computing support, and external computing training participation in training.

Third, TAM neglects the conditions, environment, and context that promote the perceived usefulness and perceived ease of use it IT adoption. Marangunić and Granić [58] indicated that a field with considerable potential is the study of various information systems and environments. Nevertheless, more efforts to examine various environmental factors, including emotion, habits, personality differences, and technology change, are necessary. Recently, more research has focused on facilitating conditions or structural constraints in TAM. For example, Ngai, Poon, and Chan [59] investigated facilitating conditions and showed how they affect technology acceptance. Venkatesh and Bala [46] elaborated on facilitating conditions that are related to individuals’ control beliefs regarding the availability of organizational resources and support structures to facilitate system use. They also found that perceived enablers or barriers in the environment influence a person’s perception of ease or difficulty of performing a task [60]. Ajibade [61] argued that IT experiences promote the ease of use of technology, while technology acceptance and intention is moderated by more structural constraints like the company’s rules, policies, and IT guidelines. Similarly, Lin and Wu [62] confirmed the role of intra-and extraorganizational factors as causal factors of end-user computing perception.

Following previous research, this study utilizes the extended TAM to explain the adoptive behavior and influencing factors of the fourth industrial revolution in governments. This study seeks to validate TAM in research on emerging new technology movement by analyzing factors that influence adoptive behavior in the central government of South Korea. Generally seen as a forerunner in the adoption and implementation of IT policies, South Korea is driving IT-based policy initiatives toward the successful transformation of the fourth industrial revolution [11]. Therefore, South Korea provides a useful field to carry out this study.

There have been many debates on the definition and concept of the fourth industrial revolution because of variation in countries’ focus on applications and content [11,21]. As the application of core components in the fourth industrial revolution requires a comprehensive understanding of its application and support from various stakeholders, the role of public employees is especially critical. For successful implementation, it is necessary to develop appropriate operating systems and institutional arrangements to increase adaptability and flexibility in various polices, especially complex fields like environment and public health. In this regard, the current study applies institutional theory to the basic TAM for a better understanding on the impact of external arrangements that are embedded in individuals’ usage of new technology. Because the interplay between technology usage and social contexts is complex and recursive, institutional aspects of IT contribute significantly to technology selection and use [63]. Institutions strongly influence peoples’ procedural decisions in the workplace [64] and employee behavior because their work procedure contents and standards are guidelines for the application of decision rules.

### 2.3. Hypothesis Development

TAM and extended ITAM posit complex human behaviors and decision-making processes that are subject to human–computer interaction. Researchers should take into account these intertwining relations in the model. This studies examine not only perceptional factors about institutional ones. A neoinstitutional framework makes links between the individual/behavioral and social/contextual sides of technology acceptance. Neoinstitutional theory applies its theories to explain technology implementation in an institutional environment. It regards informationization as a kind of institutional change and a process of institutionalization [65,66]. Jepperson ([67], pp. 145–152) defined an institution as “a social order or pattern that has attained a certain state or property”. Institutionalism is “a theoretical strategy that features institutional theories and seeks to develop and apply them”. According to institutionalism, institutions constrain and exert on an organization and organizational factors [68,69]. DiMaggio and Powell [68] suggested the three institutional pressures in organization: coercive, normative, and mimetic pressures. Similarly, Scott [69,70] proposed three institutional pillars (regulative, normative, and cultural-cognitive) that constrain and normalize individuals’ behavior. Institutions then “represent constraints on the options that individuals and collectives are likely to exercise, albeit constraints that are open to modification over time” ([69], p. 94).

In an information system (IS) context, institutional approaches focus not only on “how institutions influence the design, use, and consequences of technologies, either within or across organizations” ([71], p. 153) but also how they have an impact on users’ behavior in IS. Institutional theory sets the importance of contextualizing IT within the wider socioeconomic and political landscape [66]. Institutionalism focuses on the impact of institutions on technology users. Institutionalists believed in the formative power of institution and context in organization [72,73], which influences the way people think and constrains the way they act. Ciborra and Lanzara ([74], p. 70) describe formative contexts as “the set of institutional arrangements and cognitive imageries that inform the actors’ practical and reasoning routines in organizations”. Jensen et al. [75] addressed the electronic patient record (EPR) implementation works at three levels: the organizational field, the organizational/group, and the individual/sociocognitive level. In particular, macro-level structures, as well as individual interpretations, influence the adoption of IS. In the course of adoption, the institution significantly influences individual perceptions. Beglaryan et al. [52] examined the barriers of information system implementation concern and found that there are not only individual level factors: (a) perception, (b) expectancy and (c) utility of IS; however, there are also more institutional factors: (a) financial, (b) structural, (c) technical barriers (lack of infrastructure and suboptimal nature of the applied technology and solutions), (d) unavailability of facilitating conditions (involvement, training, organizational support, technical and expert support, and (e) lack of legal framework in IS implementation processes.

In the TAM model, institutional factors are regarded as external or social process elements. For example, Igbaria et al. [57] demonstrated that exogenous variables such as management support and external support have a significant impact on both perceived ease of use and perceived usefulness. Social influence processes (subjective norm, voluntariness, and image) and cognitive instrumental processes (job relevance, output quality, result demonstrability, and perceived ease of use) significantly influenced user acceptance [24]. Although institutions are not directly discernible in TAM, several variables are made available for attributions for institutions. For example, Venkatesh and Davis [24] identified significant antecedents such as subjective norm, perceived behavioral control, and self-efficacy. Subjective norms are shared collective beliefs that are regarded as an institution in institutionalism [76].

TAM also focuses on culture, which is a type of institution. The cultural-cognitive approach of neoinstitutional theory emphasizes the role of shared beliefs and ideas in shaping individual and organizational behaviors ([52], p. 52). When Straub et al. [77] compared the TAM model across three different countries, they found that TAM holds for both the United States and Switzerland but not for Japan, implying that TAM may not predict technology usage across all cultures. Beglaryan et al. [52] showed that administrative monitoring with an institutional character has a positive influence on perceived usefulness. Among the various institutional frameworks of TAM, culture is one of the challenging contexts that affect technology adoptive behavior [78]. Culture is shared with people who live within the same social environments. It consists of formal and informal rules of social interactions [79]. Therefore, culture may play a critical role in reducing uncertainty or risk avoidance behavior when confronting new technology. For example, individuals with the same culture who scored high on the uncertainty avoidance dimension may tend to seek ways to reduce risk and heavily rely on institutional arrangements [80].

However, with the wide and different ways of defining culture, it is difficult to examine and measure culture in an observable and constant manner to all individuals with the same cultural backgrounds [81]. Therefore, researchers usually regard culture as a type of collective institution or embedded institutional arrangement [82]. In this regard, McCoy et al. [81] suggested that the TAM model was considered to fit well in several countries, but some individual variations appeared as well. Thus, the TAM model might depend on culture as an institution collectively. Individual differences in the same cultural backgrounds, on the other hand, would be investigated for further consideration. Based on this discussion and previous studies, it can be assumed that institutions affect individuals’ thinking and behavior, suggesting the following hypothesis.

**Hypothesis** **1A.** *Institution is positively related to the perceived usefulness (PU) and the perceived ease of use (PEOU) of new technology in the fourth industrial revolution*.

As organizations and their members generally adapt to and operate in institutional arrangements (e.g., laws, orders, procedures, and instructions), institutions influence employees’ behavior and define appropriate behavior in these practices [64,77]. As March and Olson ([83], p. 22) have argued, the institution defines the “logic of appropriateness of behavior” that clarifies appropriate behavior and the individual’s role. Therefore, institutions regulate as a means of interlinking policy goals with individual actions [55]. Because institutions define responsibilities and goals, it is necessary for employees to accept institutional logic and applicable behaviors. This logical mechanism creates organizational and social conditions for the adoption of new technology in the workplace. The behavior of public employees can be more bounded by external institutions, such as laws and formal government procedures that consist of complex rule systems [84]. Thus, we attempted to develop an extended TAM model by adding institutional arrangement variables to further understand new technology adoption behavior of employees. Based on these arguments, we propose the following hypotheses to test the aforementioned logic.

**Hypothesis** **1B.** *External institution is more positively related to the perceived usefulness (PU) and the perceived ease of use (PEOU) of new technology than internal institution.*

The perceived ease of use is the degree to which the person believes that using the particular system would be free of effort [23,54]. Davis [23] originally hypothesized that the user’s attitude toward the information system was a major influential factor in whether the user would actually accept or reject the system. The attitude of the user was considered to be influenced by perceived usefulness and perceived ease of use. Subramanian [85] showed that perceived usefulness, not ease of use, is a main determinant of predicted future use of information systems. In addition, Chau [86] found that ease of use has the largest impact on specific software acceptance; perceived usefulness, not perceived ease of use, was found to be a significant determinant of attitude and intention for technology use. Igbaria et al. [57] indicated that perceived ease of use is a main factor in explaining perceived usefulness and system use. However, although perceived ease of use significantly predicts intention to use, the explanation power is secondary, following perceived usefulness [23].

**Hypothesis** **2.** *Perceived usefulness (PU) is positively related to the intention to use new technology in the fourth industrial revolution.*

Davis [23] suggested that “ease of use operates through usefulness” ([23], p 332). According to Davis [54], perceived ease of use has a direct influence on perceived usefulness. Davis [23] demonstrated that perceived ease of use fully mediated the effects on use intentions of perceived output quality. Davis et al. [87] also explained that perceived ease of use generally affects IT adoption indirectly through its effect on perceived usefulness because perceived ease of use is instrumental in making new IT more useful. Legris et al. [88]’s meta-study reported that the correlation between perceived ease of use and use intention had a significant positive relationship in 16 of 28 studies. However, Gefen and Straub [89] comment that the role of perceived ease of use in TAM remains controversial in that some studies show that perceived ease of use does directly affect either self-reported use or intended IT use.

**Hypothesis** **3A.** *Perceived ease of use (PEOU) is positively related to the intention to use new technology in the fourth industrial revolution*.

Davis [23] defined perceived usefulness as the degree to which a person believes that using the particular system would enhance her/his job performance. Igbaria et al. [57] and Abdullah et al. [90] showed that perceived usefulness is a variable that directly affects the intention to use an information system. When comparing the explanation of perceived usefulness and perceived ease of use, Davis et al. [23] reported that the perceived usefulness had 50% more impact than ease in determining information system use. Similarly, Keil et al. [91] demonstrated that perceived usefulness is a more important variable than ease of use in influencing system use. Legris et al. [88]’s meta-study showed the relationships between perceived ease of use and perceived usefulness demonstrated statistical significance in 21 of 28 studies.

**Hypothesis** **3B.** *Perceived ease of use (PEOU) is positively related to perceived usefulness (PU).*

According to Venkatesh and Bala [46]’s TAM 3 study, perceived ease of use and perceived usefulness play mediating roles in the relationship between their determinants and outcome factors. In particular, since perceived ease of use and perceived usefulness have different antecedents, the roles of both are very different, and therefore, they have differentiation and uniqueness as a path of influence. Abdullah et al. [90] showed that perceived ease of use and perceived usefulness mediated the relationships between external variables and students’ intention to use the e-portfolio. However, Beglaryan et al. [52] showed that perceived usefulness plays a mediating role between administrative monitoring and intention to use, but perceived ease of use does not perform such a function. They explained that this is because the perceived ease of use directly affects perceived usefulness and plays a different role as a variable.

**Hypothesis** **4.** *Perceived ease of use (PEOU) and perceived usefulness (PU) positively mediate the relationship between institution and intention to use new technology in the fourth industrial revolution.*

The following equations represent the proposed hypotheses. The baseline model posits that the adoption of new technologies is a function of the institutional space that surrounds the organization as well as individual elements.

Based on these assumptions, the structural model was constructed to test the logic and our hypotheses.

PEOU = ƒ(institution),(1)

PU = ƒ(institution, PEOU),(2)

Intention to use = ƒ{institution, PEOU, PU, M(PEOU, PU)}(3)

where:

- *Institution* includes laws, regulations, guidelines, strategies, and work procedures
- *Intention to use* is the level of individual’s behavioral intention to adopt new technologies
- *M(PEOU, PU)* plays a mediating role in explaining intention to use

**Figure 1:**
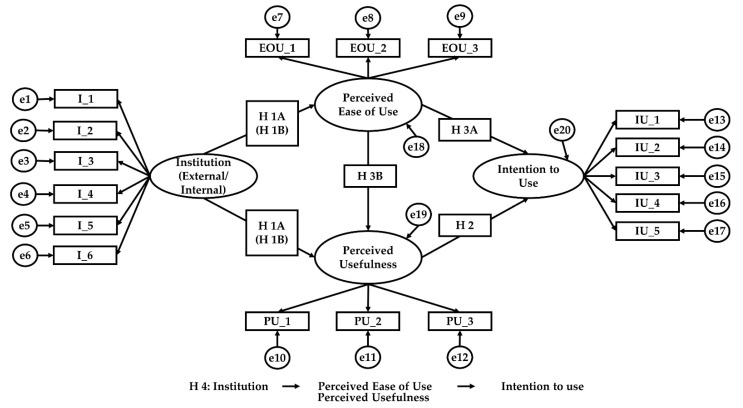
Conceptual and hypothesized model for new technology adoption during the fourth industrial revolution.

Figure 1 shows the conceptual model and proposed relationships between the variables. In Structural Equation Modelling (SEM), latent variables are not directly observed but are inferred by the covariation among a set of observed variables (also called reflective indicators). SEM combines factor analysis and regression, which provides far greater flexibility to the modeler than either of these two analysis methods. This is distinct from doing a factor analysis and then inputting the factor scores into a multiple regression.

The variables in ellipses represent latent constructs that are explained by the reflective indicators depicted with rectangles. In our proposed model, latent variables IU_1 through IU_5 stand for the items in the test for intention to use and e13 through e17 stand for measurement errors (unreliability) in each item. In addition, SEM considers measurement error by modeling it explicitly when estimating latent variables from indicators [92]. These error terms are depicted with circles.

At the center of the model lies the assumption that perceived ease of use and perceived usefulness have mediating roles that have an effect on attitudes and behavioral intentions. The model suggests that the indirect pathway, from institution to behavioral intention to use, is determined by a set of personal beliefs that influence usage behavior.

As the purpose of our study is to suggest a constructive artifact of digital informatics of human–computer interaction, it is worthwhile evaluating our proposal in terms of information system perspective [93,94]. Information system research should analyze the interplay between information technology policy, organizational architecture and contexts, and user behavior [94]. In this regard, our conceptual focus is on the interplay between organization-based artifacts and technology adoption behavior. We seek to build a unified model for the development of design and behavioral science [94,95]. To evaluate the appropriateness of our proposal, we tried to apply the guidelines and principles of information system research because our study is an integrative attempt between behavioral science and design science research [95]. Hevner et al. [93] suggested the guidelines and recommendations that include design of an artifact, problem relevance, design evaluation, research contributions, research rigor, design as a search process, and communication of research. The guidelines and principles of information systems by Hevner et al. [93] and its adequacy to this study are outlined in Table 1. Although it is difficult to address all the guidelines [95], we tried to apply these criteria in our proposed model to identify the important considerations for developing a proactive framework while understanding the requirements in planning for new information technology in the fourth industrial revolution in various countries. However, even though our proposed model mostly fulfills these criteria, there are a few guidelines that are partially adequate. Thus, there are partially adequate fulfillments for our model according to Henver et al. [93]’s guidelines.

New technology in the fourth industrial revolution is highly based on interactions among individuals, technologies, organizations, and people. Therefore, it is critical to investigate the causal factors to adopt and use IS. In an IS, technology and behavior are not mutually exclusive [96,97]; research in IS is often categorized into two frameworks: behavior and design science [93]. Because our study is trying to find new digital informatics, the conceptual model is to examine the interconnection between system design factors (institution) and behavioral factors (intention to use with perceived ease of use and usefulness) to explain the adoption of new technology in the fourth industrial revolution. Our conceptual model particularly focuses on the organization and human-based artifacts that address relationships between institutions and technology adoption behavior in the fourth industrial revolution. Specifically, we introduce the concept of institutions as an enabler that can facilitate the usage behavior of new technology. It is critical to analyze institutional structures in operational processes and policy implementation because of the fundamental role of the institution as a driving factor for new technology adoption behavior. Since our model suggests a more extended IT adoption model that is broadly applicable in relevant settings, it can extend the scope of research on information system and design science. It can also contribute to top managers of organizations by suggesting technology-based solutions to solve business problems. In considering the guidelines and principles of information system perspectives, it can be concluded that the presented model and its implications will provide further explanation for IT adoption phenomena in the field of artifacts that solve important organizational problems.

## 3. Data and Methods

### 3.1. Research Method

TAM is widely applied across various fields in IS research. Many researchers have tried to provide an extension of TAM by integrating contextual and external factors. To offer a deeper explanation of technology adoption behavior, both empirical testing of the hypothesized model and investigating new influencing factors are needed. Qualitative research can provide in-depth understanding of IT adoption; however, exploring subjective experiences and subtle aspects of behaviors is a more effective way to learn the underlying reasons and motivations for IT adoption. This study focuses on the growing and recent phenomenon of TAM application in the latest industry techniques. Therefore, a quantitative research approach was chosen for this study. SEM was employed to examine our proposed hypotheses because it is the preferred method of testing a series of complex and multiple relationships constituting a set of an entire theory [92,98]. In SEM, the simultaneous effects of direct and indirect relations between variables are reflected. In addition, subjective variables such as people’s attitudes are often inaccessible to direct measures. SEM ties multiple observed measures to the substantive latent variables and takes account of measurement errors in observed measures. SEM can appropriately reduce measurement errors of constructs by estimating error variance parameters for the latent variables. Therefore, it is a suitable method to test complicated causal relationships between human perceptions and attitudes measured by multiple indicators. Compared to common quantitative methods, such as correlation and regression, the strength of SEM is that researchers can specify an a priori relationship between variables [99,100]. Researchers can test whether hypothesized relationships derived from theory are reflected in the sample data [92]. In SEM, two approaches are widely used: covariance-based SEM (CB-SEM) and partial least squares SEM (PLS-SEM). CB-SEM adopts a common factor model approach, which calculates covariance between the variables, and only that variance is included in estimating the construct measures that can be explained by the common factor (one unobserved variable) and individual random error. In contrast, PLS-SEM utilizes all the variance of independent variables that explains the variance of dependent variables. CB-SEM is preferred for theory confirmation and theory testing, while PLS-SEM is primarily used for exploratory research and hypothesis development [101,102,103,104]. In addition, CB-SEM is recommended when the sample size is large (more than 100). In this study, the CB-SEM method (specifically, maximum likelihood estimation) was chosen because our conceptual model was based on the established TAM theory and the sample size is 300.

The number of samples is a critical issue in SEM. Bentler and Yuan ([105], p. 181) explained that the most natural method for analyzing non-normal data, the asymptotically distribution-free procedure, is not defined when the sample size is less than the number of nonduplicated elements in the sample covariance. Bentler and Chou [106] believe that the number of cases needs to be five times the number of free parameters. On the other hand, Jöreskog and Sörbom [107] state that if the measured variable is less than 12, 200 cases are required, and if the number of measured variables is 12 or more, 1.5q (q + 1) equation is applied, where q represents the number of items that are used in the analysis. Mitchell [108] suggested the rule of thumb that there should be 10 to 20 times as many cases as variables. Similarly, according to Stevens [99], 15 cases per measured variable or indicator are needed. We added this discussion to the method section of the paper.

### 3.2. Data

To address the research questions and hypotheses, our study used a dataset from an open archive of the Korea Institute of Public Administration (KIPA). The KIPA is one of the leading public research organizations funded by the central government that mainly focuses on administrative developments and practices affecting public employees. Various surveys on policy and practical issues were exclusively conducted by KIPA to support government policymaking and implementation. Thus, datasets from the surveys are provided by formal request and review for further research [109]. According to the regulations of the KIPA on consent ownership and usage, it has been authorized for use in our research. The independent variables, mediating variables, dependent variables, and control variables used in this study were measured through the survey data on the perception of new technology in the fourth industrial revolution.

The survey into the state of new digital technology in the fourth industrial revolution was designed to identify primary factors that influence the adoption of new technologies in the public sector. The survey was conducted by KIPA through an online web page from 5 to 28 September 2018. Public employees who work in IT services in the central governments of South Korea were the target population of the survey. Random and cluster sampling methods were used for data collection. A total of 333 target respondents were randomly selected from employees of IT service divisions from 18 central government ministries, which are regulated by the Government Organization Act of South Korea. The candidates were contacted by phone prior to the survey to ensure that they agreed to participate in the survey. A website address was sent to them via e-mail and respondents logged onto the website to conduct the online survey; 300 respondents participated in the survey (90.09% of response rate). The total population size of the survey was 132,422 and our sample size was 300 with 95% confidence internal and 5.6% error. Determining an appropriate sample size for SEM is debatable where there is no prevailing consensus. Many guidelines have been suggested for the optimal sample size, such as the N: q rule [110] which uses the ratio of observations to estimated parameters, where N represents the number of cases and q represents the number of parameters that should be estimated. In this heuristic approach, the recommended sample-size-to-parameters ratio ranged from 5:1 to 20:1 [106].

It depends on not only the complexity of the model but also additional factors (e.g., the number of parameters, normality of the data). CB-SEM requires a larger sample size than PLS-SEM [92], and the median sample size is about 200 cases in studies where maximum likelihood estimation is used [111]. Barrett [112] also suggested that SEM analysis with a small sample (*N* < 200) may be problematic. Given these requirements, even though archival data was used, we concluded that 300 participants was appropriate to test the conceptual model.

The survey sample reflects the proportion of the total number of central government public employees and each proportion by ranking. Table 2 shows the characteristics of the respondents. The hierarchical position of respondents ranged from 3rd (managers) to 9th (lowest-level employees). Respondents included managers (grades 3–4), middle-managers (5–7), and team members (8–9, and others). In terms of gender, 71.7% of the respondents were male and 28.3% were female.

### 3.3. Measures

The questionnaires used in this study were designed by KIPA [109]. The items were derived from validated measurements that were used and verified in previous TAM research. To ensure the construct and content validity of the measures, items selected for the variables in this study were adapted from the KIPA reports. Multiple items were used for all of the measures in order to improve reliability and validity.

**Figure 2:**
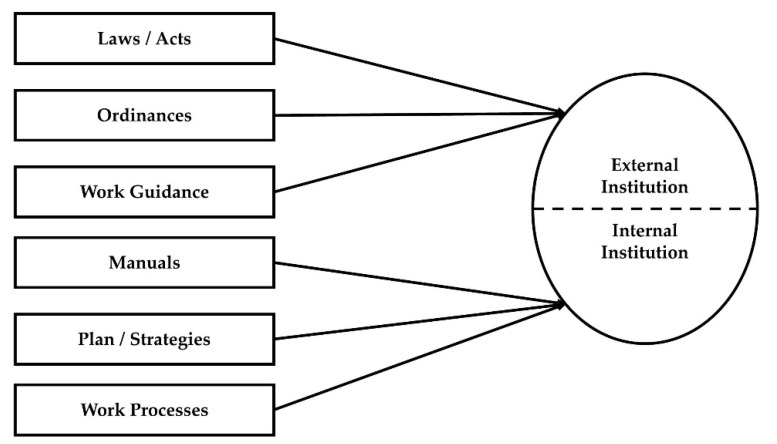
The measurement and components of institution.

*Institution.* Institution can be conceived as laws, norms, and systems that confine the way people select, implement, and use IT in public organizations [70,113]. Public officials are restrained by not only laws but also administrative rules, manuals, or plans that governmental organizations accept. Therefore, broader aspects of general rules and regulations should be adequately addressed in this study. In this sense, we used indicators of “institution” that were developed by KIPA to evaluate the level of administrative innovation using the fourth industrial revolution technology in government services. The concept of institution used in this study consists of six components shown in Figure 2. Respondents were asked to rate the degree to which they agreed or disagreed with the following statement: “There are laws/acts, ordinances, work guidance, manuals, plans/strategies, and work processes that can facilitate the use of the fourth industrial revolution technologies in the administrative service delivery.”

Perceived ease of use related to computer self-efficacy, perception of external control, and computer playfulness [23,46] were measured using a five-point Likert scale. Perceived usefulness (PU) refers to subjective norm, image, job relevance, output quality, and result demonstrability [23,46]. Intention to use’s measurement focused on purpose to use, motivation to use, and sustainable effort to use [47,51].

*Perceived ease of use*. Perceived ease of use was measured by seven items adapted from previous research by Venkatesh and Bala [46] who suggested TAM3. However, four items (computer anxiety, perceived enjoyment, objective usability, and compatibility) were excluded due to low factor loadings. As a result, the determinants of perceived ease of use in this study consisted of three items: computer self-efficacy, perception of external control, and computer playfulness. Computer self-efficacy refers to individuals’ beliefs about their ability to use and control a computer system. Perception of external control refers to individuals’ beliefs about sufficient organizational and technological support for facilitating and adopting new technology in their organization. Computer playfulness refers to intrinsic motivation and willingness to use any new system.

*Perceived usefulness.* Perceived usefulness (PU) was measured by five items adapted from the study by Venkatesh and Bala [46]. They suggested five types of determinants of PU: subjective norm, image, job relevance, output quality, and result demonstrability. Subjective norm refers to “the degree to which an individual perceives that people who are important to him/her think one should or should not use the technology” ([46], p. 277). Image refers to “an enhancement of one’s social system” ([114], p. 195). Job relevance refers to the applicability of technology in one’s work. Output quality refers to “the degree to which a person believes that the technology performs a job’s tasks well” ([24], p. 191). Result demonstrability refers to a tangible, observable, and communicable improvement of work outcome caused by the use of technology.

*Intention to use*. Behavioral intention predicts and facilitates actual usage behavior. Respondents were asked questions about their attitude toward the use of new technologies (such as artificial intelligence, internet of things, and big data). Intention to use was measured using three items that refer to purpose and motivation to use new technologies and intention to put effort into using new technologies.

### 3.4. Analysis Method: Structural Equation Modeling

We use SEM as main analysis method. In terms of the statistical approach, SEM has good advantages for testing a series of complex and simultaneous relationships. SEM is a multivariate technique used to test all coefficients (relationships) in the complete model simultaneously. Simple ordinary least squares (OLS) regression cannot assess the significance of particular relationships between variables when moderating effects are hypothesized. In SEM, path coefficients (also called connection strengths) are parameters of the structural model and are estimated through a series of algebraic manipulations. The maximum likelihood fit function is used to calculate the path coefficients which are iteratively modified [107]. The path coefficient represents the change of the dependent (responding) variable according to a unit change of explanatory variable when all other variables in the model remain constant. The path coefficients are similar to b (unstandardized coefficient) or beta (β, standardized coefficient) in the regression model. The standard error (SE) of the coefficient (or standard deviation of the estimate) is taken into account in order to indicate how the estimated sample means precise an unknown mean of a population. This allows researchers to calculate a confidence interval that provides the range of observed effect size or *p*-value that assesses whether the estimates are significantly different from some reference value.

The purpose of SEM is to specify a model derived from theory and the estimation of the parameters of the model is derived from the conventional approach of SEM. The overall objective of the conventional approach is to connect the theory and specification of the model. Within this approach, exploratory factor analysis and confirmatory factor analysis are performed to test the proposed model. At this stage, the measurement model is estimated and the structural model can be estimated later. The full model can be structured through a continuous model modification and evaluation of goodness-of-fit until the model meets the criterion of adequate fit. This is discussed in more detail in the data and methods chapter. The meaning and interpretation of parameters in the SEM are outlined in Table 3.

## 4. Results

SEM consists of two powerful statistical approaches: exploratory factor analysis (EFA) and structural modeling where structural path analysis is applied. EFA is used to identify the number of hypothetical factors or latent variables that can be explained by covariance among a set of observed variables. Therefore, a priori specification of the number of variables is not required in EFA [111,115]. Instead, the structural model (also referred to as confirmatory factor analysis (CFA)) is designed to confirm priori conceptualized and hypothesized models; therefore, the exact number of variables should be specified in order to conduct CFA [116]. First, as a primary test, we conducted Harman’s single factor test by loading all variables onto a single factor without a rotation using SPSS. 12.0 [117]. Harman’s single factor test is a widespread statistical technique to identify common method bias. It is designed to test whether one component will explain more than 50% of the covariance among the measures. If common method variance exists, one factor accounts for the majority of the covariance between the variables. Results showed that a common latent factor explained 30.24% of the covariance, which suggested that there was no common methods bias.

The construct validity of measures was investigated in two steps: EFA and CFA. The characteristics of these two steps are outlined in Table 4. EFA based on principal component analysis with varimax rotation was conducted. Exploratory factor analysis, also referred to as the unrestricted factor model, is a multivariate statistical method used to uncover the underlying structure of measures and identify latent variables [92,111,115]. Common factors among measured variables are identified while the structure and correlations among these observed variables are explained. In EFA, varimax rotations are used to enhance the interpretability of retained factors and clarify the relationship among factors. In this step, potential factors were affirmed on theoretical backgrounds and the basic construct was developed according to the following results. Items with lower factor loadings were extracted (the lowest factor loading was 0.588) and a total of 17 items were selected for the measurement model. In EFA, all observed measures depend on all factors, which were not specified by researchers beforehand; thus, EFA may generate several possible structures and models, from a one-factor model to multiple-factor model [11,111,115,116]. As a result, four factors were derived and the cumulative percentage of variance explained by them was 65. The Bartlett’s test of sphericity was performed to identify whether the variances were equal for all samples. The chi-squared value for the test was high and significant (approximate χ^2^ = 2498.853, *p* < 0.0001). The Kaiser-Meyer-Olkin (KMO) measure of sampling adequacy was 0.849, rejecting the null hypothesis that there was no difference in variances between the groups [107].

### 4.1. Measurement Model

We followed the two-step procedure proposed by Anderson and Gerbing [119], which consists of a measurement model and a structural model. In this stage, after obtaining initial instruments, we developed a measurement model and performed a CFA using AMOS 18.0. In CFA, each variable is derived from the factors specified by EFA and the researcher’s theoretical expectation; that is, restricted measurement models are identified and analyzed [111]. SEM allows researchers to evaluate relationships among variables comprehensively by providing a transition from EFA to CFA. The restricted model reflects a priori hypotheses and knowledge of the theory, which in turn allows researchers to test the relationship between observed variables and underlying latent constructs. Six common model-fit indicators were used to assess the goodness-of-fit of the measurement model. As presented in Table 5, all fit indices exceeded the recommended threshold. The comparative fit index (CFI), goodness of fit index (GFI), normed fit index (NFI), and Tucker–Lewis index (TLI) were all above 0.90. The standardized root mean residual (SRMR) was less than 0.08 [107], and the root mean square error of approximation (RMSEA) was less than 0.08 [120].

Contrary to EFA, the exact number of factors should always be defined in CFA before analysis. However, CFA does not necessarily confirm the initial restricted model that fits the data. In this case, the modified hypotheses and specified models should be proposed as alternative models [111]. In this study, CFA confirmed the hypothesized restricted model and initial constructs derived from EFA. The results of the CFA are presented in Table 6. All selected items had an acceptable value of factor loadings above 0.5 [120,121]. In addition, average variance extracted (AVE) was calculated in order to assess discriminant validity. AVE shows the level of variance that is captured by a construct compared to the level of variance due to measurement error. The values of AVE of all four factors were greater than the suggested threshold of 0.5 [112]. Overall, the measurement model indicated reasonable and acceptable convergent validity and discriminant validity.

### 4.2. Reliability Analysis and Correlations

Table 7 summarizes the descriptive statistics, the measure of scale reliability (Cronbach’s alpha), and the result of correlation analysis between the variables. The values of Cronbach’s alpha for all the factors were above the criterion of 0.70 [121], which means that scale items had high internal consistency. In addition, we calculated Pearson’s correlation coefficient (*r*) in order to evaluate the association between the variables. As shown in Table 7, correlations between the factors were statistically significant and showed predicted directions in our hypotheses. The dependent variable, intention to use, was positively related to institution (*r* = 0.276, *p* < 0.01), perceived usefulness (*r* = 0.551, *p* < 0.01), and perceived ease of use (*r* = 0.557, *p* < 0.01).

### 4.3. Hypothesis Tests

#### 4.3.1. Structural Model

**Figure 3:**
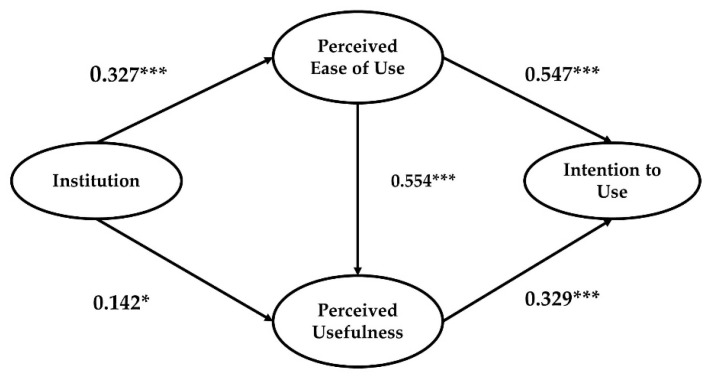
Results of the baseline model. Note: *N* = 300. * *p* < 0.05, *** *p* < 0.001.

The structural model was identified in order to test the hypotheses. Beginning with the hypothesis tests, multiple fit statistics were examined for model fit. As shown in Table 5, the results showed a reasonably good model fit, supporting the conceptual model we suggested (CFI = 0.957; GFI = 0.924; NFI = 0.917; SRMR = 0.058; and RMSEA = 0.056). Thus, we could proceed to an analysis of the path coefficients. First, we focused on the standardized direct effect of predictor variables on the response variables in the model. As Figure 3 outlines, overall results were in the predicted directions in accordance with the aforementioned hypothesis. Results showed that institutions had a positive and significant effect on perceived ease of use (β = 0.327, *p* < 0.001) and perceived usefulness (β = 0.142, *p* < 0.05), in support of Hypothesis 1A. This suggests that institutions can play an important role in the adoption and diffusion of new technology, supporting previous studies that focused on the institutional perspective [122,123]. Institution explained 10.7% of the variance in perceived ease of use (squared multiple correlations or *R*^2^ = 0.107). In terms of Hypothesis 2, the effect of perceived usefulness on intention to use was positive and significant (β = 0.329, *p* < 0.001). The results also showed that perceived ease of use had a strong and positive impact on intention to use (β = 0.547, *p* < 0.001), supporting Hypothesis 3A. The results indicate that those who perceive that the fourth industrial revolution technology is useful and simple are more likely to have a higher intention to use it. The path coefficient between perceived ease of use and perceived usefulness was positive and statistically significant (β = 0.554, *p* < 0.001), in support of Hypothesis 3B. In terms of perceived usefulness, 37.8% of variance in perceived usefulness was explained by predictor variables (perceived ease of use and institution).

SEM also allows one to examine the total effect of each independent variable on any dependent variable. Total effects are calculated through the decomposition of effects, where the direct path coefficient between the two variables and the indirect effect between the two through other mediating variables are summed [124]. Total effects consider the mediating effects of the intervening variables that might potentially influence the direct effect. In doing so, total effects can provide an understanding of which factors are more important in determining the level of dependent variable. The indirect effect of institutions on intention to use through perceived ease of use and perceived usefulness was tested using the bootstrapping method. The indirect effect was positive and statistically significant (β = 0.116, *p* < 0.01) in support of Hypothesis 4. In addition, the total effect of institution on (Institution→ perceived ease of use→ intention to use) and (Institution→ perceived ease of use→ perceived usefulness→ intention to use) was positive and significant (β = 0.267, *p* < 0.01) (Table 7). The result is not surprising given the body of work in technology studies that asserted intention to use is shaped both by perceived usefulness and perceived ease of use. We focused particularly on the impact of institutions and lend support to the positive relations in TAM paths. The summary of the hypothesis test results is presented in Table 8 and Figure 3.

In order to explore more specific institutional settings and to further establish the measure of institution, we additionally adopted two-factor institutional models. The focus of the alternative models is the dimensions of institutions: whether they are created and enforced by entities inside or outside the organization.

By adopting externally legitimized formal frameworks and internal administrative process, organizations may strengthen the engagement of their internal members in change and innovations [125]. It is noted that the adoption of new technology frequently implements similar practices in the environment without clear usefulness [126]. In this respect, the diffusion of change within the organization is influenced by external forces; formation and establishment are influenced by internal forces [127]. Institutions enforce diffusion from the top down, while individuals are immersed in innovation from the bottom up. Considering this perspective, it can be assumed that institutional features might influence new technology adoption differently in the fourth industrial revolution. Thus, we are trying to examine the influence of specific settings in institutions. Institutional change often arises in the integration of top-down and bottom-up frameworks; it requires synthetic efforts toward technological change and innovation [128].

**Figure 4:**
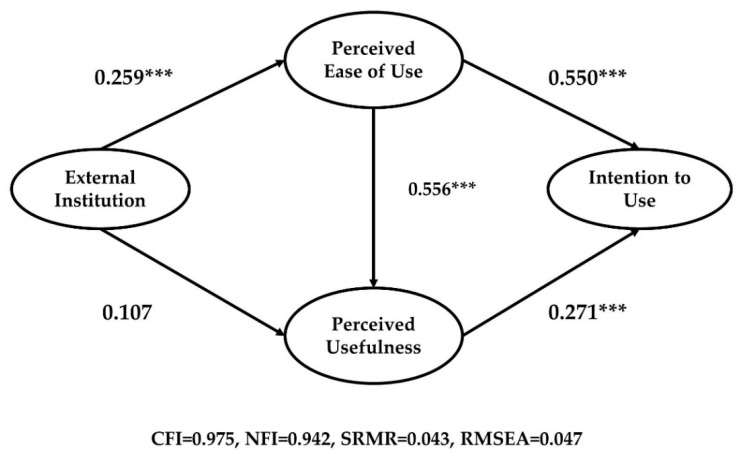
Results of the external institution model. Note: *N* = 300. *** *p* < 0.001.

An attempt to compare alternative models lets us not only review the variety of institutional systems but also determine the superiority of the conceptual modes. These two dimensions generated two-factor institutional models illustrated in Figure 4. External institutions (laws, acts, and ordinances) are enacted by legislators who can exercise political control over the operation of public organizations. Regarding path dependence and feedback frameworks, external institutions have a strong effect on early moments in new technology adoption. Because of their strong effect, these external institutions might bring inflexibility and rigidness in utilization of new technology: just follow the paths [129].

Internal institutions (work guidance, manuals, and plans/strategies) are implemented by the top of the bureaucratic hierarchy as a means of agency management. As the interrelations between organization and institution are complicated and diverse, actors in organizations tend to choose institutional suboptimal solutions or arrangements to reduce uncertainty and ambiguity in the implementation process [130]. Therefore, we tried to examine external and internal institutional influence on the intention to use new technology in the fourth industrial revolution.

**Figure 5:**
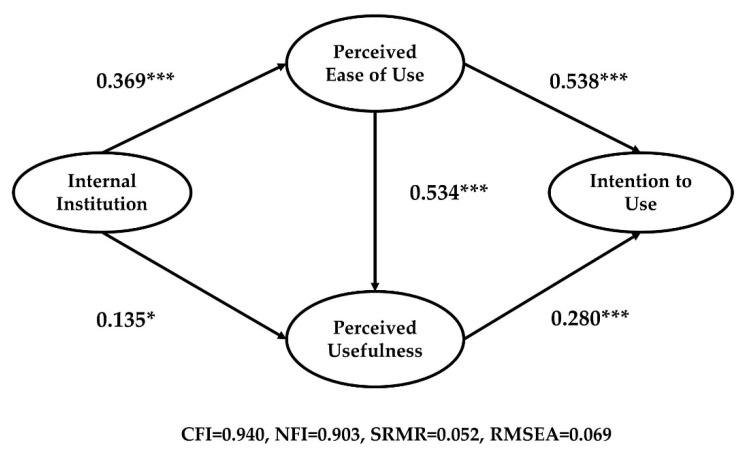
Results of the internal institution model. Note: *N* = 300. * *p* < 0.05, *** *p* < 0.001.

Our results showed that the directions of path coefficients adhere to the general TAM hypotheses. In terms of model fit, the external institution model showed adequate fit (CFI = 0.975, NFI = 0.942, SRMR = 0.043, RMSEA = 0.047) and the internal institution model also showed adequate fit (CFI = 0.940, NFI = 0.903, SRMR = 0.052, RMSEA = 0.069). In the external institution model (Figure 4), external institutions had a positive and significant effect on perceived ease of use, and the standardized total effect on intention to use was 0.291 (*p* < 0.01). Regarding the internal institution model (Figure 5), perceived ease of use and perceived usefulness had positive and significant relationships with internal institutions. However, the internal institution explained less variance of dependent variables than did the external institution. The standardized total effect of internal institution on intention to use was 0.210 (*p* < 0.01). The findings may indicate that institutional arrangements made by external entities can be more powerful than managerial actions when adopting new technologies in the public sector, therefore confirming our Hypothesis 1B.

Regarding the characteristics of organizational structure, it should be considered that there are possible differences in institutional influences between organizations with high and low hierarchical levels. Because our study was based on a high hierarchical organization (central government), it is worthwhile to discuss possible differences when we apply our two-factor institutional models to a low hierarchical organization (for example, a public expert organization). Regarding organizational members’ tendency to reduce uncertainty in the decision-making process [131], the members in low hierarchical organization might show the same results of institutional influences when adopting new technologies.

However, in terms of an organization’s complexity, centralization, and formalization, it would be considered that the influence of internal institutions would have a strong effect on new technology adoption in low hierarchical organization [132,133]. Because a low hierarchical organization has fewer managerial layers, organizational members could easily participate in the decision-making process, especially when making internal institutions like manuals and operational standards. Therefore, when adopting new technologies, internal institutions would show strong influence unlike high hierarchical organizations. Furthermore, hierarchical levels represent the system of authority for coordinating tasks and ordering the specialization of functions. These systems use varied amounts of hierarchy and control to shape behaviors; decisions are made from above and organizational members mainly receive orders. Therefore, when adopting new technologies, external institutions have strong impacts with well-defined links in the chain of command in high hierarchical organizations. Meanwhile, low hierarchical organizations have relatively few links; they might more focus on their internal institutions than external [134]. Therefore, it is possible to assume that the result of two-factor institution models in our study might be different if we apply this model in low hierarchical organization.

#### 4.3.2. Alternative Expanded Model

**Figure 6:**
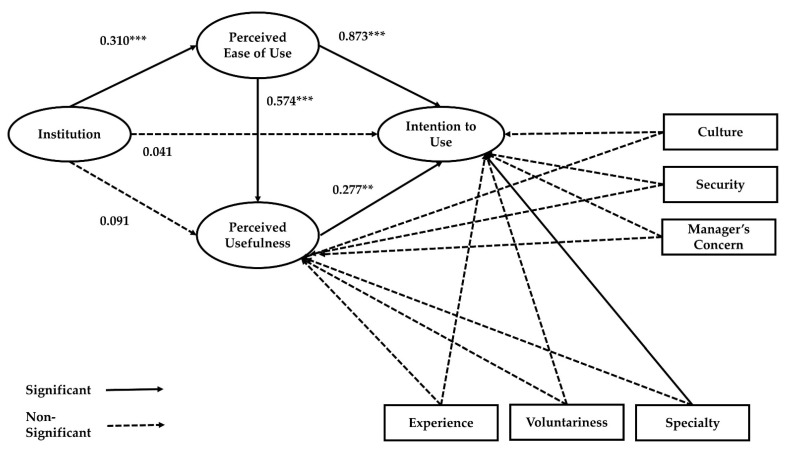
Alternative expanded model for the acceptance of new information technology. Note: *N* = 300. ** *p* < 0.01, *** *p* < 0.001.

In SEM studies, the standard procedures provide a baseline model based on general theory and test a modified model based on possible relationships between other indicators [99]. During the process, theoretical support and key conceptual assumptions of the model should be maintained. Researchers can change paths or variables and observe the coefficient and model fit changes compared to other models. This can be done by adding new variables or removing problematic latent variables. All steps need to be backed up with logical reasoning and theory. After several iterations of paths and variables, we decided to use the alternative expanded model, explained in Figure 6.

In doing so, we tried to give a more comprehensive explanation of complex human behaviors and attitudes that are constrained by various conditions and circumstances. The purpose of examining an additional alternative expanded model becomes clear when considering the fundamental nature of technology diffusion and adoption in organizations. Technology adoption and diffusion occur as a continuous process based on a series of individual decisions that are mostly calculated by the incremental benefits of using the technology. In addition, technology adoption is usually requested by a top-down management and often seen as an investment decision made by managers [70]. Researchers suggested that technology adoption occurs in stages according to individual differences. It is often modified by environmental conditions and uncertainty [71].

Therefore, the alternative expanded model posits that contextual factors may have direct and indirect effects on intention to use. In order to give a more comprehensive explanation of complex new technology phenomena, we constructed an alternative expanded model and tested non-mediated models. The proposed baseline model only considered causal path relationships of institutions, perceived ease of use, and perceived usefulness to intention to use of new technology in the fourth industrial revolution. To examine the influence of individual factors, we included intrinsic factors (experience, voluntariness), organizational factors (culture, security), and task-related factors (specialty, manager’s concern) [46,134]. Among individual intrinsic factors, experience might be important for adopting new technology [135]. New ways of collecting and sharing digital information have already been established by digital-based ubiquitous technologies, users who are accustomed to these trends might easily accept new technology. Thus, experience may be important for adopting new technology [136]. The degree to which prospective users perceive their decision is voluntary is important as it can affect when they adopt new technologies [56]. When technology adoption is organizationally imperative, user motivations vary and certain users restrain such type of mandate [128]. In this respect, voluntariness may affect adoptive behavior of new technology.

When introducing new technologies, the ability for individuals to adapt and modify their patterns is challenged. In this respect, a strong cultural foundation will facilitate the acceptance and success of new technologies [14,137]. Hence, culture can have an effect on new technology adoption. Because of the importance of new technology for social and economic growth, as well as an increasing focus on data collection, privacy has become a public concern [138]. It is critical to find the right balance between technology deployment and data security with user privacy, especially in the public domain [139]. Consequently, privacy can have an effect on new technology adoption.

New technology in the fourth industrial revolution might influence the types of job tasks required of individuals, which health professionals during the current pandemic have experienced [140]. Thus, we tried to include task-related factors (specialty, manager’s concern) in the alternative expanded model. In recent technological advances, including social networking, smartphones, sensors, and embedded systems, different attitudes and skills have been required [141], which has added to the burden of some employees. Consequently, specialty might have an effect on new technology adoption. Regarding managers’ organizational role, manager behavior has been discussed as one of the most significant factors in adopting new technologies [142]. Managers can assist in the technology adoption process by advising all users in the organization [143]. In this respect, managers’ concern will impact the adoption of new technology in the fourth industrial revolution.

Therefore, we examined the possible impact of individual features (experience, voluntariness, and specialty) and organizational conditions (culture, security, and managers’ concern). Individual experience was measured by “I have experienced public services provided by new technologies in the fourth industrial revolution (such as artificial intelligence, internet of things, and big data)”. Voluntariness was measured by “I think it is important to foster public service innovation using new technologies in the fourth industrial revolution”. Specialty was measured by “I have job specialties so that I can adopt new technologies in the fourth industrial revolution”. Organizational conditions were measured by the following: “My organization is trying to foster a culture of new technology in the fourth industrial revolution utilization”, “My organization provides technical security measures to protect IT-enabled public services”, and “Managers in my organization try to foster IT-enabled public services innovation.”

Our approach to the expanded modification model with individual and organizational factors can contribute to the development of information system research. From the perspective of “good design science” [93], studies must be presented effectively to both technology-oriented and management-oriented audiences. In this regard, results must be addressed to both the technology scholars and management communities with adequate objectivity and significance. Following this standard, a modified model can provide a thorough discussion of the behavioral processes for new technology adoption and managerial actions that are necessary. Our study results can provide useful solutions for managers and employees when they face obstacles to adopting new technology.

We tested whether significant improvement in model fit was made when adding new variables [92]. In order to investigate the differential effects of each condition and to compare model fits among the proposed models, changes of path and variables were added in stages from Models 1 to 4. In the first modification, a direct relationship between institution and intention to use was presumed (Submodel 1). The second modification was made to the structural model by adding individual factors (Submodel 2). The third modification was made by adding organizational factors to the baseline model (Submodel 3). In the final modification in Model 4, both individual and organizational factors were added and tested. Overall model fit indices are presented in Table 9.

Generally, the proposed alternative models were acceptable; however, the model fits were not greatly improved over the baseline model. Thus, we concluded that alternative models may not be preferable to the baseline model. Although alternative models did not appear superior in terms of model fits, it should be noted that more conditional variables were considered and the simultaneous effects were examined.

Beyond these model fit results, significant path coefficients were found in most of the expected directions and the results supported the verified causal relationships of the baseline model. Table 9 represents the total effects of each variable on intention to use and Figure 6 only represents direct effects between the variables. The total effect is combined with direct and indirect effects. In SEM, the pathway from the exogenous latent variable to endogenous outcome variable through the mediator calculates indirect or mediating effect. As shown in Table 10, the significant and positive total effects of institution and perceived ease of use were maintained in all of the alternative models. More importantly, coefficients of both variables were raised incrementally as alternative factors were added, thus confirming the positive relation between institution and technology adoption behavior. This also suggests that the impacts of institution and perceived ease of use on intention to use remains dominant when other individual and work conditions are controlled. In addition, the direct effect of institution on intention to use did not approach significance in Figure 6; however, the total effect was significantly mediated by perceived ease of use and perceived usefulness. This suggests institutions can affect intention to use only through mediating effects of perceived ease of use and perceived usefulness. Again, perceived ease of use and perceived usefulness had full mediating effects on the relationship between institution and intention to use. Perceived ease of use also had a significant and positive effect on intention to use in Submodel 2 and Submodel 3; however, significance was not found in the full Submodel 4. Of the three predictors, the improvement of the coefficient of perceived ease of use was noticeable compared to other variables, indicating that perceived ease of use is relatively more important in determining an outcome. Thus, it may be concluded that institutions strengthen the level of intention to use mainly through perceived ease of use. The summary of the hypotheses test results are as follows.

As with any previous TAM studies, the effects of both perceived ease of use and perceived usefulness on intention to use were positive and significant in the basic parsimonious model. Furthermore, institutions significantly affect perceived ease of use and perceived usefulness. Institutions have a significant and strong indirect influence on intention to use, which would lead one to expect that institutional factors may have a crucial role in new technology acceptance of public employees in South Korean central government agencies. The test results of the alternative models lend support to the important role of institutions as an external facilitating factor, thus confirming Hypothesis 1. While the direct effects of institutions on perceived usefulness did not show significance in submodel 4, its total influence on intention to use was still reinforced by mediating variables. In terms of the total effects of contextual variables, specialty and security showed a negative and significant effect on intention to use in submodel 2 and 3, respectively. However, direct effects were not significant (Figure 6). In submodel 4, only specialty had a significant and negative effect on institution (total effect) and the direct effect was also significant as shown in Figure 6.

## 5. Discussion and Implication

### 5.1. Discussing the Main Results

Our study aimed to analyze the links between the impact of institutions, perceived ease of use, perceived usefulness, and intention to use of new technology adoption in the fourth industrial revolution [11,12,20,31,144]. By using individual-level data from a survey of public employees in the Korean central government conducted in 2018, this study empirically examined the important factors for adopting new technology. The understanding and recognition of public employees engaging in new emerging technology is important because of their role in decision-making and value creation in collective issues like the environment and public health [145,146].

The contributions of our study to the healthcare field are suggested as follows: First, the usage of digital technology and developments in the healthcare field are expected to increase significantly following COVID-19. Regarding the role of information systems in COVID-19, this study implies that institutional environments should be considered when initiating digital health related policies or projects. After tracking websites’ digital communication strategies in Latin America hospitals during the COVID-19 pandemic, Tejedor et al. [147] suggested that digital media could constitute legitimate resources for healthcare information consumption, so their accuracy and proper development seem to be significant to becoming a genuine source. In addition, after studying Electronic Health Record (EHR), Beglaryan et al. [52] indicated that institutional factors as well as personal benefits at the micro level should be considered. In particular, they suggest that the critical role of social factors, like institutions, hinders success of informatization at the organizational level. Moreover, Ahmad et al. [148] found that social influence had a positive impact on patients’ continued intention to use digital health wearables. These studies suggest not only utility-based managerial factors but also social structural ones should be considered in investigating new technology adoption. While it is important to emphasize the convenience and benefits of operationalization of new technologies, it is also important to construct basic institutional arrangements for their utilization. The usage of information technology for pandemic management is still a long way from achieving an integrated system focused on effective cooperation between humans and machines for disease prevention and treatment [28]. As legal frameworks have been critical to more promptly responding to the COVID-19 crisis, the need for more comprehensive regional and national disease registries is required. It is also emphasized that coordination between agencies and organizations is required for successful testing and quarantine for individuals with COVID symptoms using new technology such as contact tracing [139]. However, without clear guidance and recommendations, successful coordination among stakeholders is difficult to achieve. Therefore, the results of this study suggest that institutional adjustments are required to promote the acceptance of new technology, especially during crisis like COVID-19.

Second, our findings suggest that institutions had an indirect impact on the intention to use new technology of the fourth industrial revolution. It is important to consider what factors affect government employees’ intentions to use new technology as they introduce and organize various collective strategies on public issues such as pandemics. The institutions do not ensure that the actual application of emerging technologies can occur without the mediation of presumed ease and usefulness. As a result, when we introduce new technologies, we must understand how people actually perceive the ease of use and usefulness of such technologies. In particular, the COVID-19 pandemic involved huge demands that necessitated the collection and analysis of digital health data using new technology. As a result, the role of government employees is critical. Thus, it is worthwhile to investigate the factors of new technology adoption, especially when it is intended to be used by public employees confronting this unprecedented issue.

Third, when considering the design of information systems for healthcare services, it needs to consider whether external and internal institutions had a differential effect on the intention to use new technology [128]. In the results of our study, in terms of uncertainty reduction, the effect of internal institutions is stronger. As a consequence, we would conclude that when implementing new technologies, it is important to provide employees with a standardized procedure and best practices [149]. However, external institutions had a stronger total effect on intention to use than internal institutions, where perceived ease of use and perceived usefulness have mediating roles in our result. Thus, we can conclude that official government schemes and systems may have superiority over managerial actions when implementing new technology. Therefore, it is necessary to provide adequate external institutions (like laws and ordinances) for effective adopting new technology in healthcare fields.

In Table 11, we summarized the contributions of our study in the fourth industrial revolution and TAM studies. This section will provide a concise description of the experimental results, their interpretation, and the experimental conclusions that can be drawn.

### 5.2. Practical Implications

Our study empirically investigated the influence of institutions on new technology adoption of the fourth industrial revolution. The characteristics of these new technological developments are complicated [21], and it is expected that the fourth industrial revolution will bring new changes by growing networking, data-based intelligence, and knowledge sharing between humans by real-time computer interaction [153]. When new technology is initiated in an organization, institutional influence is normally based on centralization; however, simply adopting external requirements could result in short-term usage of new technology like perceived ease of use but not long-term goals like usefulness and intention to use in our study [13].

New technological advancements like the fourth industrial revolution should be closely interconnected with institutional arrangements such as strategies that allow a structure to adjust or preserve stabilization after experiencing periods of instability [154]. For instance, in the case of South Korea, after the Middle East Respiratory Syndrome (MERS) outbreak in 2015, structures and processes such as strengthened legislative authority for quarantine and surveillance systems encouraged people to promptly adapt to digital technologies in response to the pandemic [155]. Thus, these institutional facilitators and the groundwork helped the new technology to be quickly used for public health. These institutional arrangements for disease control that include relative digital data management have enabled South Korea to extend accelerated testing and case recording to a greater portion of the population when faced with COVID-19 [155]. Contrariwise, the United States, where lengthy lines for coronavirus testing wrapped around the block early in the pandemic, demonstrated a defective monitoring process even several months after the pandemic outbreak.

Countries like Singapore and Australia launched a digital app and QR tracing, while data encrypted technology was reserved for government-based utilization [156,157]. In Singapore’s attempts to control the COVID-19 pandemic, technologies such as artificial intelligence and data analytics have played a critical role. The Australian experience also shows that specific institutional responses are crucial for public health prevention. For example, with the launch of a monitoring and tracing application for smartphones, COVIDSafe, the Australian federal government rapidly replaced the conventional contact tracing methods. These strong institutional adjustments increase user safety [158]; the application was downloaded by six million people in a short period.

Given the pandemic’s uncertain nature and the risks it presents, it made sense for government authorities to first centralize decision making, such as school openings, and create or implement strategies for delivering instruction to fulfill regulatory requirements [159]. Nevertheless, as we can see with cases in various countries, administrative regulatory decisions during the pandemic require unambiguous evidence of the reasons and necessities for the public. Without a large amount of obvious data from reliable objective technological procedures, public uncertainty and doubt cannot be easily alleviated. We should consider public confidence in public health officials and other policy makers. While the latest pandemic is unsettling, we can envision a worse situation in the future.

As we have seen in the previous examples of response to the COVID-19 pandemic in various countries, new technology adoption does not always lead to complete adoption and use. One of the main reasons for the failure of U.S. governance was not due to technological difficulty but rather to institutional instability and perceived utility [160]. Thus, as our study suggested, detailed considerations should be prepared when established institutions adopt new technology in the fourth industrial revolution.

## 6. Conclusions

In this study, we assessed the causal path relationships of new technology adoption in the fourth industrial revolution by an empirical survey of public employees of the central government in South Korea. Since government employees play such a significant role in the decision to construct new technology deployment [161,162], they are in a key position to introduce and utilize the advantages of new technology. The convergence of physical and digital technologies is one of the key features of the fourth industrial revolution [153] and it could open new opportunities to transform conventional human–computer interaction, as we have witnessed during the current global COVID-19 pandemic [155].

We identified that institutions influence perceived ease of use and usefulness, which mediate the impact of institutions on intention to use new technology [163]. According to our findings, institutions have a significant influence on the intention of public employees to use new technology in terms of ease of use and usefulness, which has a mediating effect on intention to use. This means that if digital-based new technology is assumed to be the new means of information usage, the behavioral patterns of new technology adoption would not be strongly differentiated from conventional technology use [23,152], except for institutional impact. In addition, as we have witnessed, the applications of new technology in the fourth industrial revolution to confront the pandemic are constantly increasing. However, without appropriate consideration of users’ perception and behavior, this technological use would not be as effective [164].

In addition, as the results of our alternative expanded model showed, institutions cannot directly impact intention to use new technology without the mediating effect on perceived ease of use and usefulness if we do not control the causal path relationship [165]. It is important to note that the casual path direction of the influence of institutions is only through ease of use, not usefulness in the expanded alternate model, implying that perceived ease of use should receive significant consideration when new technology is introduced in a society [165,166]. In conclusion, this study specified the determinants of new technology adoption in the fourth industrial revolution and, in particular, sought a causal path between institutions and the intention to use new technology with perceived ease of use and usefulness as mediators.

The findings of this study could be meaningful in many respects. However, they also present certain limitations that will provide a basis for future research. This study performed statistical tests and analyses based on cross-sectional survey data collected at a single point in time. Thus, it is not possible to use a panel analysis methodology that examines the trend of usage over time. In addition, even though we conducted several statistical tests to avoid possible methodological bias in our model, mono source bias or correlated measurement error might not be completely excluded.

Because of the contextual features of the South Korean public sector, it is necessary to consider biases from contextual factors like culture in further research [167]. Furthermore, given that our alternative two-factor institution models are based on high hierarchical level organization, the results might show differently if they were applied to low hierarchical level organization. In addition, to offer greater insight into the phenomenon of usage and utilization, the use of case studies should be considered to investigate more interpretive research of new technology in the fourth industrial revolution phenomena [13,14]. Future studies are encouraged to explore the issues and practices of new technology and its effectiveness in the context of determinants.

## Figures and Tables

**Table 1:** Guidelines and principles of information systems by Hevner et al. [93] and its application in this study.

Guidelines and Principles	Description	Application for This Study
Design of an artifact	Design science research must produce a viable artifact in the form of a construct, a model, a method, or an instantiation.	This study suggests the Institution-based Technology Acceptance Model (ITAM) as a framework to construct more effective information system arrangement and organizational settings in the fourth industrial revolution.
Problem Relevance	The objective of design science research is to develop technology-based solutions to important and relevant business problems.	This study suggests technology-based (security), organization-based (internal institution and manager’s concern), and people-based (attitude toward technology and culture) artifacts to address new technology acceptance issues.
Design Evaluation	The utility, quality, and efficacy of a design artifact must be rigorously demonstrated via a well-executed evaluation method.	The proposed model in this study is evaluated for informed argument from the knowledge base (e.g., perception of users) to explain its possible utility. It also integrates prototype IT artifacts that can be mathematically evaluated.
Research Contributions	Effective design science must provide clear and verifiable contributions in the areas of design artifacts, design foundations, and/or design methodologies.	This study and the artifact, ITAM provide both research and practical contributions to South Korea and other countries which adopt new information technology in the fourth industrial revolution. The result of this study provides institutional requirements and constraints for new information technology adoption.
Research Rigor	Design science research relies upon the application of rigorous methods in both the construction and evaluation of design artifact.	Our proposed model addresses possible alternatives for IT artifacts that can be applied to managerial and behavioral changes within appropriate environments. The applicability of the model and causal relationships between latent variables are verified by utilization of sophisticated statistical methods.
Design as a Search Process	The search for an effective artifact requires utilizing available means to reach desired ends while satisfying laws in the problem environment.	The proposed model provides effective solutions to address new technology acceptance problem. The possible solutions suggested in this study include laws, managerial actions, and technical issues which in turn provide a pragmatic approach for design science research.
Communication of the Research	Design science research must be presented effectively both to the technology oriented as well as management-oriented audiences.	The results provide both technological and managerial implications to enable the artifact to be implemented. It also provides an analytic framework for researchers as well as managers to evaluate new IT artifact.

**Table 2:** Characteristics of the respondents.

		Frequency	Percent
Position	3rd	6	2.0
	4th	34	11.3
	5th	106	35.3
	6th	71	23.7
	7th	49	16.3
	8th	2	0.7
	9th	2	0.7
	Other	30	10.0
Gender	Male	215	71.7
	Female	85	28.3
Total		300	100.0

**Table 3:** The meanings and interpretations of statistics in the SEM.

Statistics	Meaning	Interpretation
*S.E* (Standard error)	The standard error means an estimate of the standard deviation (S.D) of the coefficient.	The standard error allows us to identify the magnitude of error which is made in estimating an outcome variable from an independent variable.
Estimate (β, standardized coefficient)	The standardized coefficient is calculated by multiplying the unstandardized coefficient by the ratio of standard deviation of explanatory variable and outcome variable.	Each of the estimated parameters represents the amount of change in the dependent variable as a function of a single unit change in the explanatory variable.
*AVE* (Average Variance Extracted)	AVE is the level of variance that is captured by a construct compared to the level of variance.	The suggested threshold that is normally higher than 0.50 would be acceptable.
*R*^2^ (R-Square)	As the independent variables are correlated in SEM, the R^2^ of each estimate indicates the partial effect of each variable on the dependent variable.	The R^2^ of the structural model can be interpreted as a proportion of variance explained. The full structural model relationships between latent variables and direct variables have the highest partial coefficient.
*CFI* (The Comparative Fit Index)	CFI is an incremental relative fit index that measures the relative improvement in the fit of the researcher’s model.	CFI is a revised form of NFI. It ranged from 0 to 1. The recommended threshold is 0.9 or more.
*GFI* (The Goodness of Fit)	GFI is the proportion of variance accounted for by the estimated population covariance.	GFI indicates the proportion of variance explained by the estimated population covariance. The recommended threshold is 0.9 or more.
*NFI* (The Normed-Fit Index)/ *TLI* (Tucker–Lewis Index)	NFI indicates whether the proposed model improves the fit compared to the null model. TLI (also called non-Normed-Fit) is preferable for a small sample.	NFI ranges between 0 and 1. The recommended threshold for NFI is 0.9 or more.
*SRMR* (Standardized Root Mean Square Residual)	SRMR is the standardized difference between the residuals of the observed sample covariance matrix and the predicted hypothesized model.	SRMR ranges between 0 and 1. The recommended threshold for SRMR is 0.08 or less.
*RMSEA* (Root Mean Square Error of Approximation)	RMSEA represents a parsimony-adjusted index.	RMSEA ranges from 0 to 1. Values closer to 0 represent a good fit. The recommended threshold is 0.08 or less.

**Table 4:** The characteristics of explanatory factor analysis (EFA) and confirmatory factor analysis (CFA).

	EFA	CFA
Purpose	Determining latent variables; Developing scale [115]	Investigating model assumption; Testing validity of items [118]
Necessity	Explaining the existing structure [118]	Investigating previous proven structure; Requiring strong model assumption [116]
Procedure	Initial testing between items [115]	Following EFA, evaluating or confirming the extent [116]
Usage	Factor decision when the number of factors between items is not known; Resulting in a preliminary rather than definite outcome [116]	Prior knowledge of the expected relationships between items and factors are required [116]

**Table 5:** Fit indices for the measurement and structural models.

Fit Indices	Recommended Value	Measurement Model	Structural Model
CFI	>0.90	0.965	0.957
GFI	>0.90	0.931	0.924
NFI	>0.90	0.924	0.917
TLI	>0.90	0.956	0.947
SRMR	<0.08	0.055	0.058
RMSEA	<0.08	0.051	0.056

**Table 6:** Results of confirmatory factor analysis (CFA).

Survey Items		Factor Loadings	S.E	AVE
Institution	I_1	0.703	0.045	0.672
	I_2	0.785	0.038	
	I_3	0.837	0.033	
	I_4	0.812	0.030	
	I_5	0.655	0.047	
	I_6	0.664	0.046	
PU	PU_1	0.797	0.040	0.632
	PU_2	0.906	0.028	
	PU_3	0.548	0.065	
	PU_4	0.532	0.051	
	PU_5	0.592	0.057	
PEOU	PEOU_1	0.704	0.048	0.581
	PEOU_2	0.561	0.059	
	PEOU_3	0.714	0.050	
IU	IU_1	0.544	0.075	0.701
	IU_2	0.749	0.047	
	IU_3	0.832	0.036	

Note: PU: perceived usefulness; PEOU: perceived ease of use; IU: intention to use.

**Table 7:** Descriptive statistics, reliabilities, and correlations.

Variable	Mean	S.D	Reliability	1	2	3	4
1. Institution	3.03	0.636	0.896	1			
2. PU	3.62	0.556	0.819	0.308 **	1		
3. PEOU	3.63	0.595	0.699	0.285 **	0.409 **	1	
4. IU	3.84	0.559	0.736	0.276 **	0.551 **	0.557 **	1

Note: ** *p* < 0.01, PU: perceived usefulness; PEOU: perceived ease of use; IU: intention to use.

**Table 8:** Summary of the hypothesis test results (standardized direct effect).

Path			Estimate	Hypotheses	Test Results	*R* ^2^
Institution	→	PEOU	0.327 (*p* = 0.000)	H 1_1	Supported	0.107
Institution PEOU	→ →	PU PU	0.142 (*p* = 0.020) 0.554 (*p* = 0.000)	H 1_1 H3_2	Supported Supported	0.378
PU PEOU	→ →	IU IU	0.329 (*p* = 0.000) 0.547 (*p* = 0.000)	H2 H3_1	Supported Supported	0.624

Note: PU: perceived usefulness; PEOU: perceived ease of use; IU: intention to use.

**Table 9:** Comparison of the goodness-of-fit.

Submodels	CFI	GFI	NFI	TLI	SRMR	RMSEA
Submodel 1	0.957	0.924	0.917	0.947	0.058	0.056
Submodel 2	0.955	0.921	0.908	0.943	0.054	0.053
Submodel 3	0.961	0.924	0.912	0.949	0.054	0.049
Submodel 4	0.956	0.918	0.903	0.940	0.052	0.049

Note: The dependent variable is intention to use. Bootstrap, bias-corrected two-tailed tests used to calculate significance of the total effects. *N* = 300.

**Table 10:** Path coefficients for the alternative models (standardized total effects).

Variables	Submodel 1	Submodel 2	Submodel 3	Submodel 4
Institution	0.267 **	0.310 *	0.370 **	0.387 *
Perceived ease of use	0.694 *	0.737 **	0.885 **	1.032 *
Perceived usefulness	0.274 **	0.282 **	0.294 *	0.277
Experience		0.093		0.085
Voluntariness		0.171		0.173
Specialty		−0.192 *		−0.264 *
Culture			−0.007	−0.019
Security			−0.284 *	−0.296
Manager’s concern			−0.017	0.028

Note: The dependent variable is intention to use. Bootstrap, bias-corrected two-tailed tests used to calculate significance of the total effects. *N* = 300. * < 0.05, ** *p* < 0.01.

**Table 11:** Theoretical contributions of this study to representative previous research.

Reference	Study Aims	Our Contributions
Sung [11]	To analyze practices of the fourth industrial revolution and industry 4.0 plan in Korea, along with guidelines and recommendations; to suggest that institutional infrastructure of central governments to lead all initiatives are required	Based on the survey of public employees in Korea, our study tested the basic theoretical framework for technological adoption of the fourth industrial revolution practices with the TAM approach.
Safar et al. [12]	To examine opinions and attitudes of inhabitants of South India with a survey method; to emphasize insufficient knowledge of the fourth industrial revolution and industry 4.0 of the potential workforce and to suggest education and requalification is necessary	Our study showed the impact of institution on technology adoption of the fourth industrial revolution is critical. In our analysis, institution is a macro-level concept that includes work guidance, manual, plans, and strategies. Thus, institution deals with proper education and requalification to each section
Anton [18]	To examine the adoption of new technological processes of public employees of internal call centers with the TAM approach; to emphasize the role of previous experience of public employees on technology, and to suggest further investigation on the effect of the environmental factors is required	Following the suggestion, our study focused on the influence of institution on technology adoption, especially huge dynamic changes of the fourth industrial revolution on public employees
Baldwin [19]	To examine ICT use of public employees in New Zealand and whether technological development could comprehensively change administrative process; to suggest technology usage is not just a technical issue but managerial investment is needed	The purpose of our study is to find causal factors of new technology adoption by public employees. Our study proved that institution strongly influences TAM framework. The alternative model also showed that institution does not unintentionally relate to technology usefulness in the fourth industrial revolution.
Pfeiffer [20]	To discuss current status of the fourth industrial revolution with in-depth analysis; to suggest further investigation of actors in various sectors about the trends	Our study aimed to investigate the adoptative behaviors of technology practices in the fourth industrial revolution, with a focus on public employees for diverse analysis about the current issue.
Lee et al. [21]	To suggest various recommendations with brainstorming techniques on the fourth industrial revolution; to emphasize the role of institution in increasing creativity in organization.	Our study empirically analyzed the influence of institution with the TAM model. We showed the critical role of institution on the development of the fourth industrial revolution.
Venkatesh and Davis [24]	To examine the impact of subjective and individual factors by extending TAM to address causal antecedents; to suggest adding designing patterns and system uses for structural consideration and functional design	Following the extended TAM, our study applied significant extensive factors like culture, experience, and voluntariness on our alternative model to confirm its role in the model. For extending theoretical constructs, our model focused on the role of institution as structural prerequisite for TAM to find whether there is causal antecedent with TAM.
Reischauer [30]	To discuss and clarify the contents and identity of the fourth industrial revolution and Industry 4.0; to address various policy implications including the development institutionalization of the fourth industrial revolution for innovation	Our study empirically tested the impact of institution on technology adoption of the fourth industrial revolution by survey of public employees to confirm the role of institution is critical.
Horst and Santiago [150]	To review and discuss the role of actors in policy process in various countries; to suggest that an institutionalized platform reframed and managed by the government is necessary	Our study investigated institution as a significant factor on technology adoption and examined its influence on the technology adoptive behavior of public employees.
Liao [33]	To review and identify influential public policy and challenges by cross country comparison; to suggest various policy implications for inclusion in clear guidelines and process for policy implementation	Our study regarded institution as a composition of liability, structural formation, and procedural requirements for empirical test.
Corrocher et al. [151]	To examine the obstacles and drivers of ICT adoption by surveying IT managers in Italy; to suggest from their empirical findings that contexts, compatible standards, and information diffusion are significant. Furthermore, authors indicated the sensitivity of institutional environment is strong and critical.	Following empirical results of ICT adoption, we empirically verified whether the role of institution is still valuable in the new technology context of the fourth industrial revolution.
Fountain [130]	To explain how information technologies affects decision-making in complex organizations, especially with theoretical and qualitative approaches to the institutional perspective	Adopting the idea of basic framework, our study tried to confirm the role of institution on technology adoption with empirical results.
Verma, Bhattacharyya and Kumar [47]	To empirically examine TAM with the system characteristics of quality and belief as causal antecedents; to suggest the influence of system and integrated model are needed	Adopting the idea of system characteristics, our study included institution with TAM to analyze its impact on technology adoption of the fourth industrial revolution.
Holden and Rada [152]	To apply TAM with extensive variables of self-efficacy on attitudes toward using; to suggest that the role of external variable needs to be studied	To examine the impact of external variables, we selected institution as a significant external variable in the emerging trends of the fourth industrial revolution
Alekseev et al. [144]	To review and analyze the process of formation of Industry 4.0; to suggest possible barriers and overcoming strategies in each stage, technology usefulness would be a key factor among them	To follow their proposition, our study examined the role of technology usefulness with extensive institutional TAM by empirically tested.
Agarwal and Prasad [56]	To examine extended TAM with emotional variables like efficacy, anxiety, and managerial variables like innovativeness on the adoption of mobile-based money; to confirm significant impact of perceived ease of use in the model and also suggest the use of SEM technique to control the issues of endogeneity issues in TAM.	Following their findings to the context of the fourth industrial revolution, we regarded perceived ease of use of new technology as an important mediating factor in the model.
Luna-Reyes and Gil-Garcia [63]	To analyze e-Government failure with regard to focus on ICT perspective on case study approach, authors demonstrated the important relationships between institutions, organization forms, and technology adoption of e-Government	Based on qualitative analysis by authors, our study focused on the role of institutions on new technology adoption of the fourth industrial revolution.

## Data Availability

The data presented in this study are available on request from the corresponding author. The data are not publicly available due to regulations and guideline of data open policy according to the Korea University.
